# MBCS: A few-shot intent detection model for manual inspection records

**DOI:** 10.1371/journal.pone.0335914

**Published:** 2025-12-10

**Authors:** Mengjie Liao, Yixin Wang, Jian Zhang, Bo Li, Zhenlong Wan

**Affiliations:** 1 School of Management Science and Engineering, Beijing Information Science and Technology University, Beijing, China; 2 Beijing Key Laboratory of Big Data for Green Development Decision-Making, Beijing, China; 3 School of Computer Science, Beijing Information Science and Technology University, Beijing, China; 4 Business School, Yangzhou University, Yangzhou, Jiangsu, China; 5 National Customs Information Center, Beijing, China; Beijing Institute of Technology, CHINA

## Abstract

Addressing the bottleneck issue of low accuracy and poor generalization in cargo risk intent detection caused by annotation scarcity in manual inspection scenarios within the import-export trade supervision domain, this study proposes an intent detection model named MBCS (Multi-task Learning with BERT for Classification and Semantic Similarity Comparison), designed for few-shot scenarios. To tackle the challenges of scarce domain-specific data and inadequate text representation capabilities, the research introduces a multi-task learning framework that integrates text classification with semantic similarity comparison. By incorporating semantic contrastive learning as an auxiliary task, the model’s semantic representation capability is enhanced. Concurrently, an attention-weight-based synonym substitution strategy was introduced, replacing the highest-attention words in the sequence by integrating contextual information. Experiments conducted on real-world customs business datasets demonstrate that MBCS achieves significant accuracy improvements of over 4.19% (4.91%) in 5-shot (10-shot) scenarios, substantially outperforming baseline models. This method provides an optimized solution for intent detection tasks plagued by the annotation scarcity dilemma.

## 1 Introduction

Intent detection, also known as intent recognition or intent classification, is one of the core tasks in natural language understanding and an important branch of text classification. This task aims to accurately identify the underlying intent or purpose by analyzing the semantic information of user input (such as text or speech). Record texts generated by professionals (such as customs officers, financial auditors, doctors, etc.) during inspection processes (e.g., inspection reports, audit opinions, diagnostic records, etc.) are a type of unstructured textual data rich in domain knowledge, widely present in fields such as finance, healthcare, and cross-border regulation. These texts typically document the professional judgments and decision-making intents of reviewers in detail, making them highly valuable for understanding specialized decision-making processes.

Especially in the context of customs tax administration, business personnel need to manually inspect imported goods to assess whether there are risks associated with the commodities and identify the specific types of risks. They then document the issues identified in the goods in detail using unstructured text. Fully mining such records from historical customs declaration data not only helps build a high-quality risk knowledge base for real-time risk prevention and control but also serves as a valuable supplement for judging various elements of cross-border trade risks. However, these manual inspection records often contain colloquial expressions, abbreviations, proprietary terms, and other non-standard vocabulary with subjective nuances, making it difficult to accurately classify and fully utilize the knowledge value embedded in such texts during business data statistics and analysis.

Although intent detection technology provides an effective solution for semantic analysis of inspection records, its practical application in customs manual inspection scenarios faces the “annotation scarcity dilemma,” making it a typical few-shot learning problem. The severe shortage of data annotated by domain experts limits the performance of supervised learning methods. Additionally, the extensive presence of domain-specific terminology and special numerical codes in inspection records falls outside the knowledge scope of general-purpose pre-trained language models, making it difficult for such models to accurately capture the semantic information of the text.

In summary, this study proposes a few-shot intent detection model for manual inspection records, named MBCS (Multi-task Learning with BERT for Classification and Semantic Similarity Comparison). This study makes the following contributions:

1. It proposes a novel synonym replacement algorithm that leverages self-attention matrix weights to generate high-quality training data.

2. It introduces a semantic similarity-based contrastive learning task within a multi-task learning framework, enhancing the model’s ability to capture fine-grained textual representations both within and between classes.

3. It employs a multiple gradient descent algorithm to balance the training dynamics of the two tasks, thereby optimizing the overall performance of the encoder.

## 2 Related work

Currently, research approaches for intent detection tasks in few-shot scenarios can be broadly categorized into six types: data augmentation-based methods, metric learning-based methods, transfer learning-based methods, meta-learning-based methods, prompt learning-based methods, and active learning-based methods.

Data augmentation methods are considered effective and reliable solutions for few-shot tasks. Tesfagergish [1] and Huang [2] et al. augmented data using the ChatGPT platform and then employed other classification models to distinguish the text. The results demonstrated that this approach improved the accuracy of the classification models. Xue et al. [3] generated sentences similar to the original input text by adding specific prompts to conditional self-encoding models. Experiments on public datasets showed that this method achieved competitive results. Guo et al. [4] performed data augmentation by constructing positive and negative sample pairs, which not only expanded the dataset but also laid the foundation for subsequent class knowledge transfer—showing certain similarities with the contrastive learning approach adopted in this paper. Li et al.[5] propose a step-wise data-augmentation approach that is coupled with staged training. The method progressively augments the original data from both a global and a local perspective—at the level of the entire corpus and within sample pairs of the same class—and divides the learning process into distinct stages according to the progression depth. Experimental results demonstrate that this strategy improves model accuracy. To address the need for extensive manual annotation in joint intent detection and slot filling tasks, Dadas et al. [6] designed a set of heuristic rules to apply random mutations to training samples for data expansion. The study defined three rules: random slot replacement, random word replacement, and altering sequence order. Experimental results on the ATIS dataset indicated that this method improved the F-score in slot filling tasks. However, these methods have several limitations. The use of large language model platforms or online APIs poses data leakage risks, making them unsuitable for sensitive data in customs domains. Conditional self-encoders rely heavily on well-designed input templates to achieve desirable performance. Random word replacement often yields limited benefits, as the substituted words may occur in positions that are not critical for the classification model. Altering sequence order can be beneficial in some cases but may disrupt semantic coherence. Random deletion and insertion operations resemble introducing noise, which might enhance model generalization but contribute little to improving accuracy.

Metric learning-based methods primarily utilize sentence-level instance representations to measure similarity between examples in a metric space. To address the overfitting issue in intent detection models caused by insufficient training data, Xu et al. [7] proposed the Semantic Transportation Prototypical Network (STPN). Unlike the Prototypical Network (PN) introduced by Snell et al. [8], their approach uses word-level representations rather than sentence-level representations as input and employs the Earth Mover’s Distance as a new metric to achieve better sample matching. Furthermore, the study reformulates the few-shot classification task as an optimal matching problem, aiming to align key semantic information between samples and treating the matching cost as a similarity measure. This method improved accuracy by at least 2% compared to baseline models (5-way, 5-shot). Yang et al. [9] enabled the model to learn discriminative representations of intent keywords by comparing the similarity between two textual views of the same text. Experimental results on four public datasets demonstrated that such representations help the model accurately identify textual intent in few-shot settings. Casanueva et al. [10] employed a pre-trained siamese sentence encoder for few-shot intent detection. Their method distinguishes different categories by measuring semantic similarity between input utterances and predefined templates. This approach achieved competitive results on several public datasets, improving accuracy by approximately 20% on the BANKING77 dataset (10-shot) compared to the baseline. However, selecting representative templates remains a critical and challenging task.

In few-shot scenarios, transfer learning enables models to leverage knowledge acquired from a source task and transfer it to a target task. This technique is highly valuable due to its ability to effectively utilize existing knowledge. Maqbool et al. [11] applied contrastive learning on top of pre-trained language models to adapt to zero-shot scenarios, resulting in performance that surpassed state-of-the-art models in the field. To enhance cross-domain transfer and generalization capabilities, Yu et al. [12] designed a meta-adapter integration algorithm that combines adapter modules to constrain model parameters and depth, while employing meta-learning to fine-tune the adapters. Experimental results demonstrated that this approach outperforms existing mainstream models on standard text generation evaluation metrics and effectively mitigates common challenges in cross-domain transfer, such as catastrophic forgetting, task interference, and training instability. To this end, Wang et al.[13] proposes a deep-transfer-learning approach that leverages cross-platform knowledge to classify imbalanced, small-scale online-consultation data on civil disputes. By jointly matching distributions and conditioning feature mappings, the method mitigates the adverse impact of outlier samples on transfer learning.

Meta-learning, as a key technique for addressing few-shot problems, enables models to rapidly adapt to new tasks with limited samples by equipping them with the ability to “learn how to learn”. Bhathiya et al. [14] applied this approach to the joint task of intent detection and slot filling, demonstrating that meta-learning can acquire prior knowledge from similar tasks in highly resource-constrained settings, thereby facilitating fast inference on target tasks. Bertinetto et al. [15] proposed a novel meta-learning framework for few-shot classification that leverages a differentiable closed-form solver to optimize model parameters. This solver directly provides analytical solutions without relying on iterative optimization processes. Han et al. [16] integrated meta-learning with adversarial domain adaptation to achieve efficient text classification in few-shot scenarios. Bao et al. [17] introduced a distributional feature-based representation method that enables effective textual learning through cross-category representation transfer. Wang et al. [18] utilized the multi-dimensional feature processing capability of 3DCNN to construct an improved prototype generator. Experimental results showed average improvements of 4.90%, 4.53%, and 8.81% on the Clinc150, Hwu64, and Liu57 datasets under (10-way, 5-shot) and (15-way, 5-shot) settings, respectively. The PaAT model [19] further enhanced accuracy by generating prototype representations through attention mechanisms between category texts, effectively addressing the long-range dependency limitations of 3DCNNs.

Prompt learning transforms classification tasks into masked language model (MLM) cloze tasks through carefully designed templates, effectively leveraging the internalized knowledge of pre-trained language models. Schick et al. [20] proposed manually crafted prompt templates to convert classification tasks into MLM-based cloze problems, enabling more efficient utilization of implicit knowledge in pre-trained LMs. Gao et al. [21] combined automatic prompt generation with dynamic example selection to enhance model performance in few-shot settings. Zhang et al. [22] integrated prompt learning with meta-learning to mitigate the need for large amounts of meta-training data, and validated the effectiveness of their approach through visual interpretation. Zeng et al. [23] designed a set of task-specific prompt templates that map the labels predicted by the pre-trained language model to existing risk labels via answer engineering. Experimental results demonstrated that this prompt-based approach outperforms fine-tuning methods. Feng et al.[24] propose a novel prompt-based continual-learning method, TIPS, that couples a two-level prompt-selection strategy with adaptive weight sets to improve selection accuracy via sparse joint tuning. Results show that the approach maintains stable prompt-selection accuracy across multiple incremental-learning sessions.

Active learning-based methods selectively choose data for annotation and have been shown to achieve performance comparable to training on full datasets while using only half the amount of labeled examples [25][26]. Farfan-Escobedo et al. [26] initially trained a model on a subset of data and then employed an active learning algorithm to select the most valuable samples from the remaining unlabeled pool for annotation, thereby improving model performance. Results demonstrated that this approach significantly reduces the amount of data required for training. Xiang et al.[27] propose PromptAL, a hybrid active-learning framework that quantifies how much each unlabeled point contributes to aligning the current empirical distribution with the target distribution, thereby refining the decision boundary. Specifically, PromptAL first leverages unlabeled data to construct sample-aware, dynamic soft prompts that shift the model’s predictive distribution and decision boundary. Subsequently, guided by the adjusted boundary, it combines uncertainty estimation with both global and local diversity to select high-quality samples that better represent the target distribution. PromptAL achieves superior performance against nine strong baselines. Shim et al. [28]propose an enhanced active-learning framework with explanation-based intervention that effectively leverages annotators’ domain knowledge during query selection. Through this framework, annotators not only understand the intrinsic reasons why each sample is recommended, but can also systematically steer the query-selection process by expressing their expertise in the form of feature weights. Experiments demonstrate that the framework remains effective in typical scenarios where the dataset contains noisy features. However, unlike the previously mentioned methods, this algorithm requires an additional pool of unlabeled data for selection, which slightly differs from the conventional few-shot learning setting.

In summary, improving the quality of text representations and enhancing data diversity are key to boosting the performance of few-shot intent detection models. To address the challenges of scarce domain-specific data and insufficient text representation capability, this study proposes a multi-task learning framework that integrates text classification with semantic similarity comparison. By introducing semantic contrastive learning as an auxiliary task, the framework strengthens the model’s semantic representation ability, thereby enhancing the performance of the encoder’s vector representations. Meanwhile, to tackle the issue of limited data diversity, the study innovatively proposes an attention-weighted synonym replacement strategy: key words are identified by analyzing the model’s attention distribution, and synonym substitution is performed under semantic constraints. This strategy effectively increases data diversity while ensuring the semantic consistency of generated samples, thereby improving the model’s generalization capability.

## 3 Materials and methods

### 3.1 Overall architecture

**Fig 1:**
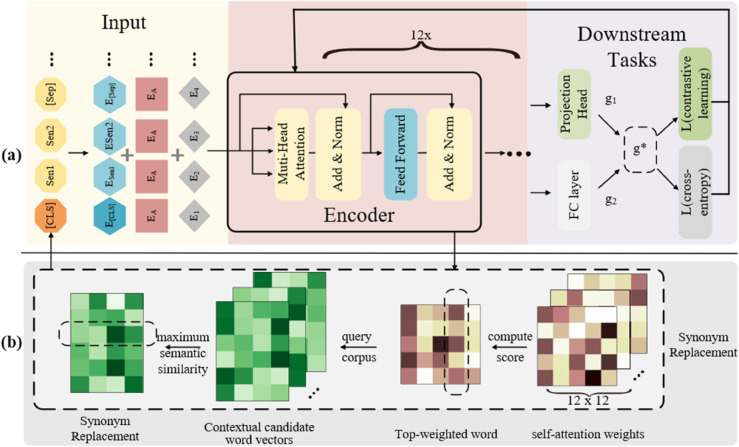
MBCS model architecture.

To address key challenges in few-shot intent detection—such as limited generalization capability and insufficient semantic representation learning caused by scarce training data—this study proposes a multi-task learning-based joint optimization framework named MBCS (Multi-task Learning with BERT for Classification and Semantic Similarity Comparison). As illustrated in Fig 1(a), MBCS adopts a hierarchical architecture consisting of: 1) An input layer responsible for preprocessing raw text and feature initialization; 2) An encoder layer that constructs deep semantic representations using a pre-trained language model; 3) A task branch layer that enables collaborative optimization through parallel execution of the main text classification task and an auxiliary semantic similarity comparison task. This architecture significantly enhances the representational capacity of the encoder via knowledge transfer across multiple tasks.

Input Layer (Fig 1(a), Input Module): This layer converts raw text into numerical representations understandable by the model. For each input text, it performs tokenization and adds special tokens. The tokenized sequence is then mapped to token IDs from the vocabulary and converted into embedding vectors.

Encoder (Fig 1(a), Encoder Module): Comprising 12 layers, the encoder processes these embedding vectors to capture contextual relationships between tokens. Through multiple stacked encoder layers, the model captures increasingly complex and fine-grained features.

Task Branch Layer (Fig 1(a), Downstream Tasks Module): This layer consists of two sub-tasks—text classification and semantic similarity comparison—both sharing the same encoder. The semantic similarity comparison task enhances the encoder’s ability to learn discriminative textual representations by contrasting semantic vectors within the same class and differentiating those from different classes, thereby supporting the main text classification task. The projection head reduces vector dimensionality. The text classification task serves as the main objective, determining the intent category. It is followed by a fully connected layer that outputs a probability distribution over classes based on the encoder’s representations. This study leverages the Multiple Gradient Descent Algorithm (MGDA) to find a common gradient direction that stabilizes the training process for both tasks.

Additionally, the study incorporates semantic enhancement through a semantics-constrained synonym replacement strategy. By computing attention matrix scores, the most attended words in the sequence are replaced with synonyms of the same part of speech from a corpus, generating new sequences for data augmentation. As illustrated in Fig 1(b), the augmented samples are combined with the original data to retrain the model.

### 3.2 Fine-tune BERT

#### 3.2.1 Pre-trained language model BERT.

The pre-trained language model BERT [29] learns rich linguistic representations from large-scale corpora by introducing the Masked Language Model (MLM) and Next Sentence Prediction (NSP) tasks. In the MLM task, randomly masked tokens in the input text are predicted by the model, enabling it to learn contextualized word representations. The NSP task, which involves determining whether two sentences are consecutive, helps the model grasp relationships between sentences. This pre-training strategy allows BERT to model both token-level and sentence-level semantic information, thereby capturing complex linguistic structures.

In intent detection tasks, BERT first tokenizes the input text into subword units using the WordPiece algorithm. This approach effectively handles out-of-vocabulary (OOV) words while reducing the size of the vocabulary. A special classification token [CLS] is added at the beginning of each input text to represent aggregated information of the entire sequence. For sentence-pair tasks, a separator token [SEP] is used to distinguish between the two sentences. In single-sentence tasks, the [SEP] token is appended at the end of the text.

The model retrieves token IDs corresponding to the text $T\in {\mathbb{R}}^{1\times k}$ by looking up the vocabulary, where *k* denotes the sequence length after adding special tokens. If the input sequence exceeds the model’s maximum supported length *n*, it is truncated; if shorter, padding tokens [PAD] are appended to reach length *n*, resulting in the final textual representation $T^{\prime }\in {\mathbb{R}}^{1\times n}$. The input vector $X\in {\mathbb{R}}^{n\times d}$ is obtained by summing three different types of vectors, as shown in Eq (1):

$$𝐄={𝐄}_{token}+{𝐄}_{segment}+{𝐄}_{position}.$$ (1)

Among these, ${𝐄}_{token}$ are derived from the subword units; ${𝐄}_{segment}$ indicate which sentence each token belongs to, distinguishing between different sentences; and ${𝐄}_{position}$ are incorporated into each token to provide the model with an understanding of the sequential order of the input.

The encoder serves as the core component of the BERT model. Taking the BERT-base model as an example, its encoder consists of 12 Transformer encoder layers stacked in sequence. Each layer primarily comprises two sub-layers: a multi-head self-attention mechanism and a feed-forward neural network. The output of each layer undergoes residual connection and layer normalization to stabilize the training process and accelerate convergence. The input vector $X\in {\mathbb{R}}^{n\times d}$ is processed by the multi-head self-attention mechanism, producing a context-aware representation matrix $Z\in {\mathbb{R}}^{n\times d}$. The output *Z* incorporates interaction information between each token and all other tokens in the sequence, enabling it to capture global contextual relationships. After the multi-head self-attention mechanism, the input vector *X* is added to the output Z of the self-attention module, as shown in Eq (2). The result of this residual connection, denoted as ${𝐙}_{res}$, is then normalized via layer normalization to obtain ${𝐙}_{norm}$.

$${𝐙}_{res}=𝐗+𝐙.$$ (2)

$${𝐙}_{norm}=({𝐙}_{res}).$$ (3)

After residual connection and layer normalization, the output of the multi-head self-attention mechanism, denoted as ${𝐙}_{norm}$ , is fed into the feed-forward neural network. The feed-forward network processes the representation of each token independently to further extract features.

The feed-forward neural network consists of two fully connected layers with a ReLU activation function in between. The computational process is in Eq (4):

$$\mathrm{FFNN}({𝐙}_{norm})=\max(0,\;{𝐙}_{norm}W_{1}+b_{1})\;W_{2}+b_{2}.$$ (4)

Here, *W*_1_ and *W*_2_ are learnable weight matrices, and *b*_1_, *b*_2_ are bias terms. The input and output of the feed-forward network are again processed through a residual connection and layer normalization, ultimately producing the output of the entire encoder. This output then serves as the input to the next encoder layer, iteratively refining the feature representation of the input text.

#### 3.2.2 Fine-tuning.

Fine-tuning refers to the process of adapting a pre-trained model to a specific task by training it on a small amount of task-specific data. As a transfer learning strategy, it leverages knowledge acquired from large-scale pre-training to achieve strong performance on smaller datasets, thereby reducing training costs and enhancing the model’s generalization capability. In text classification tasks, assuming the parameters of the BERT model are denoted as *θ*, which are initialized using pre-trained parameters $\theta_{pretrained}$, the goal of fine-tuning is to minimize the loss function *L* on the target task to update the model parameters:

$$\theta^{*}=\arg\min_{\theta }\sum_{(x,y)\in D}ℒ(f(x;\theta ),y).$$ (5)

Here, *x* denotes the input sample, *y* the ground truth label, and *D* the dataset of the target task.f(x,θ) represents the BERT model parameterized by *θ*. *L* is the cross-entropy loss function. The study employs full fine-tuning, optimizing all layers starting from $\theta_{pretrained}$. The update process is defined in Eq (6), where η denotes the learning rate:

$$\theta \leftarrow \theta -\eta \;\nabla_{\theta }ℒ(f(x;\theta ),y).$$ (6)

In the joint task of text classification and semantic similarity comparison, the study employs a multi-task fine-tuning approach where both tasks share underlying parameters. The optimization objective is defined in Eq (7), with *T* denoting the number of tasks and $\lambda_{\text{t}}$ representing the weight assigned to each task. Details of the specific loss function will be introduced in Sect 3.4.

$$\theta^{*}=\arg\underset{\theta }{\min}\sum_{i=1}^{T}\lambda_{t}\sum_{(x_{t},y_{t})\in D_{t}}{ℒ}_{t}(f(x_{t};\theta ),y_{t}).$$ (7)

### 3.3 Semantics-constrained synonym substitution

Research on Data Augmentation via Synonym Substitution. Compared to other methods such as back-translation or generative adversarial networks, this approach better preserves semantic consistency and retains core meaning in data rich in domain-specific terminology. Unlike random word replacement, this method identifies the top-n words that receive the highest attention in a sequence by comparing weight scores in the model’s self-attention matrix—words that are likely critical to classification outcomes. It then substitutes these words with same-part-of-speech synonyms that have the highest similarity scores based on corpus statistics. Experimental results demonstrate that, compared to random substitution, this method enables models to more effectively learn discriminative features within sequences. Below is a detailed introduction to the method.

The multi-head self-attention mechanism is a core component of BERT, enabling the model to dynamically focus on different parts of the input sequence during text processing, thereby capturing rich semantic information. It projects the input vectors **x** into query, key, and value vectors using trainable weight matrices *W*_*Q*_,*W*_*K*_, and $W_{V}$, respectively, as shown in Eqs (8) to (10).

$$Q=XW_{Q},$$ (8)

$$K=XW_{K}$$ (9)

$$V=XW_{V}.$$ (10)

For each word in the input sequence, the attention score with every other word (including itself) in the sequence is computed via a dot product operation, resulting in a vector of similarity scores. As shown in Eq (11), the query vector ${𝐪}_{i}$ (a row in *Q* ) of each word is multiplied with the key vector ${𝐤}_{j}$ (a row in *K* ) of every other word through dot product. The result is then scaled by a factor to prevent excessively large dot product values that may lead to gradient vanishing issues, where $\sqrt{d_{k}}$ denotes the dimension of the key vectors. The resulting scores represent the attention degree of the word toward all other words.

$$Score(q_{i},k_{j})=\frac{q_{i}⬝k_{j}^{T}}{\sqrt{d_{k}}}.$$ (11)

Taking the BERT_base_ model as an example, it consists of 12 encoder layers, each containing 12 attention heads. Each head corresponds to a self-attention matrix and independently computes attention scores, which reflect the correlations between different positions in the input sequence. Different heads focus on distinct aspects: some pay more attention to syntactic structures, while others emphasize semantic relationships. The multi-head self-attention mechanism linearly concatenates the scores computed by the different attention heads, as shown in Eq (12):

$$Mutihead(Q,K,V)=Concat(head_{1},head_{2},...,head_{h})W_{O}.$$ (12)

where *W*_*O*_ is the output weight matrix and *h* is the number of attention heads. In the self-attention matrix, each row represents the attention scores that a word pays to all other words, while each column indicates the attention scores that a word receives from all other words. In this study, the BERT model was first fine-tuned on a specific corpus using multi-task learning, which enhanced the encoder’s ability to capture discriminative features of sentences for classification. Experiments revealed that attention heads in the middle layers are more adept at capturing such classification-related features in sequences. Based on the column-wise scores of the attention matrix (excluding [CLS] and [SEP] tokens), the nouns, adjectives, or verbs that received the highest attention were identified. These words were then replaced with same-part-of-speech synonyms selected from a manually constructed corpus, rather than through manual substitution.

During the synonym replacement process, a word often has multiple candidate synonyms. To select the most suitable replacement, the study calculates the cosine similarity between word vectors and filters the candidate most semantically similar to the target word for substitution. The specific procedure is as follows: each candidate word is substituted into the sequence and reprocessed through the BERT model to generate contextualized word embeddings. The cosine similarity is then computed between the two contextualized embeddings, and the candidate with the highest similarity score is selected to replace the original word, as shown in Eq (13):

$$\cos ine\_similarity(A,B)=\frac{\sum_{i=1}^{n}A_{i}B_{i}}{\sqrt{\sum_{i=1}^{n}A_{i}^{2}}\sqrt{\sum_{i=1}^{n}B_{i}^{2}}}.$$ (13)

where *n* is the dimensionality of the vectors, and *A*_*i*_ and *B*_*i*_ are the components of vectors *A* and *B* in the i-th dimension, respectively.

During actual testing, it was observed that this method has certain limitations: synonyms with different parts of speech from the target word could also achieve high similarity scores. To ensure the quality of data augmentation, a dual-validation mechanism was introduced: (1) Using a pre-trained language model to compute the semantic similarity between the original and substituted sentences, ensuring global semantic consistency; (2) Evaluating classifier confidence to guarantee that the modified samples can still be correctly classified.

When two or more keywords in the sequence were replaced, manual inspection revealed that even if the sequence passed the validation mechanism, the semantics of the sentence were somewhat compromised, or the structure became ungrammatical. This degradation increased with the number of substituted words. In our experiments, it was empirically observed that replacing 2 to 3 words per sample yielded the most significant improvement in the accuracy of the MBCS model, even when the resulting sentences were not always grammatically perfect.

### 3.4 Multi-task learning

Multi-Task Learning (MTL) is a machine learning paradigm designed to improve a model’s generalization and performance by learning multiple related tasks simultaneously. Unlike single-task learning, MTL enhances overall effectiveness by sharing representations or parameters to leverage correlations among tasks. This study proposes combining intent detection classification with semantic similarity contrastive learning, as the latter contributes to some extent in improving the model’s classification capability.

#### 3.4.1 Text classification head.

The classification head is one of the final components of the MBCS model. The BERT model extracts contextual representations of the input text through its multi-layer Transformer encoder and outputs a hidden state vector. The classification head then maps this hidden state vector to specific class labels, selecting the category with the highest probability as the predicted user intent, as shown in Eqs (14) to (16). In this study, it consists of a fully connected layer (linear layer) followed by a softmax function.

z=Wh+b, (14)

$$p_{i}=\frac{\exp(z_{i})}{\sum_{j=1}^{k}\exp(z_{j})},$$ (15)

$$\hat{y}=\arg\underset{i}{\max}\;p_{i}.$$ (16)

For a multi-class classification problem, the cross-entropy loss function is given in Eq (17):

$$L(y,\hat{y})=-\frac{1}{N}\sum_{i=1}^{N}\sum_{c=1}^{C}y_{i,c}\log({\hat{y}}_{i,c}).$$ (17)

where *y*_*i*,*c*_ is the ground truth label of the i-th sample for class c and is the predicted probability of the i-th sample for class *c*. Here, *C* denotes the total number of classes, and *N* represents the total number of samples.

#### 3.4.2 Supervised contrastive learning.

Contrastive Learning is commonly regarded as a self-supervised learning method. It treats augmented samples of the same instance as positive pairs and other samples within the same batch as negative pairs. By pulling similar samples closer and pushing dissimilar ones apart, it learns meaningful feature representations. Extensive research has demonstrated its effectiveness in significantly improving model performance.

However, a key challenge in self-supervised methods is the “false negative” problem, where a sample is incorrectly labeled as negative despite being semantically similar to the anchor. This introduces noise and can degrade model performance. Khosla et al. [30] extended the self-supervised contrastive loss to a supervised setting by incorporating label information. In their design, samples of the same class within a batch are treated as positive pairs, while those from different classes form negative pairs for contrastive learning. Experimental results showed that this method significantly outperformed the self-supervised contrastive model SimCLR in image classification accuracy.

$$ℒ=\frac{-1}{\left|p(i)\right|}\sum_{p\in P(i)}^{N}\log\frac{\exp(z_{i}\cdot z_{p}/\tau )}{\sum_{\text{a}\in A(i)}\exp(z_{i}\cdot z_{a}/\tau )}).$$ (18)

where *N* is the number of samples in the batch; *z*_*i*_, *z*_*p*_, and *z*_*n*_ denote the embeddings of the anchor, positive sample, and negative sample, respectively; and *τ* is the temperature parameter used to control the sharpness of the similarity distribution. *P*(*i*) represents the set of indices of positive samples that belong to the same class as sample *i* , excluding *i* itself, while *A*(*i*) denotes the set of all samples in the current batch. In vector space, each sentence is represented as a point in a high-dimensional space. The dot product of sentence vectors can measure the semantic similarity between them, as it represents the sum of the extent of “pattern matching” across feature dimensions. Semantically similar sentences have similar distribution patterns in this high-dimensional space, resulting in a larger dot product.

The Eq (18) measures the similarity between samples by computing the dot product of their embeddings, scaled by the temperature parameter *τ*. When *τ* is small, differences in the similarity distribution are amplified, causing the model to focus more on sample pairs with high similarity. Conversely, when *τ* is large, the distribution becomes smoother, leading the model to treat all sample pairs more uniformly. By taking the negative logarithm, the objective of maximizing the similarity of positive pairs is transformed into minimizing the loss.

In the projection layer design of MBCS, a two-layer perceptron structure is adopted to optimize the feature space and enhance the effectiveness of supervised contrastive learning. The first fully connected layer (FC1) primarily serves to improve nonlinear expressive capability. This layer maps high-dimensional features from the backbone network to a lower-dimensional space while introducing the ReLU activation function to increase the flexibility of feature representation, enabling the model to better learn complex data distributions. The second fully connected layer (FC2) further transforms the reduced-dimensional features to make them more suitable for contrastive learning tasks. Its objective is to optimize feature projection such that samples of the same class are brought closer together in this space, while samples of different classes are pushed farther apart, thereby improving the performance of the supervised contrastive loss. Through this two-layer neural structure, not only is computational cost reduced, but discriminative power in the feature space is also enhanced, effectively boosting the overall performance of the MBCS model in intent detection and contrastive learning tasks.

#### 3.4.3 Multiple gradient descent algorithm.

In this study, the classification task and the representation learning task work jointly, sharing inductive biases between them. Traditional multi-task learning methods typically minimize the sum of losses from multiple tasks. However, since the gradient directions of the two tasks may differ or even conflict with each other, it is uncertain whether each update iteration will mutually benefit both tasks, or whether some degree of mutual inhibition exists. Therefore, instead of simply combining the loss functions, the study aims to find a method that maximizes collaboration between the two tasks. To achieve this, multi-task learning is explicitly formulated as a multi-objective optimization problem, with the goal of identifying an optimal gradient direction g^*^ that balances the objectives of both tasks.

First, the parameters of the backbone network are denoted as *θ*, and the gradients of the loss functions of the two tasks with respect to the shared parameters *θ* are computed.

$$\begin{array}{c} g_{constructive}=\nabla_{\theta }{ℒ}_{constructive},\\ g_{class}=\nabla_{\theta }{ℒ}_{class}. \end{array}$$ (19)

The direction of the gradient indicates the direction of the steepest ascent of the loss function, and its magnitude reflects the rate of change along that direction. The study aims to find a new gradient direction, referred to as the optimal gradient direction, in which both tasks are optimized as much as possible, as shown in Eq (20).

$$g*=\lambda_{1}{\text{g}}_{class}+\lambda_{2}g_{contrastive}.$$ (20)

where the weights $\lambda_{1}$,$\lambda_{2}>0$,$\lambda_{1}$  +  $\lambda_{2}=1$. This ensures that the gradients of the tasks are not summed in opposing directions. The original problem is thus transformed into an optimization problem of finding the minimum-norm solution $\|g^{*}{\|}^{2}$, as shown in Eq (21). By minimizing the gradient norm, the shortest vector g^*^ is identified, whose value corresponds to a local or global minimum—i.e., the optimal solution under the current conditions.whose value corresponds to a local or global minimum—i.e., the optimal solution under the current conditions.

$$\underset{\lambda_{1},\lambda_{2}}{\min}\;{\left\|g*\right\|}^{2}=\underset{\lambda_{1},\lambda_{2}}{\min}\;\|\lambda_{1}g_{class}+\lambda_{2}g_{constructive}{\|}^{2}.$$ (21)

The above expression represents a typical convex optimization problem. The optimal weights are solved using a quadratic programming approach, with the objective function is given in Eq (22):

$$\begin{array}{c} \underset{\lambda }{\min}\;\frac{1}{2}\|G\lambda {\|}^{2}=\underset{\lambda }{\min}\;\frac{1}{2}\lambda^{T}(G^{T}G)\lambda ,\\ \sum_{i=1}^{n}\lambda_{i}=1,\lambda_{i}\geq 0. \end{array}$$ (22)

where $G=[g_{class},g_{constractive}]\in R^{d\times 2}$ is the quadratic gradient matrix,$\lambda ={\left(\lambda_{1},\lambda_{2}\right)}^{T}$. From the above analysis, the objective function is subject to both equality and inequality constraints. For such a nonlinear programming problem, the KKT conditions are applied to solve it. The Lagrangian function is constructed in Eq (23):

$$\begin{array}{c} ℒ(\lambda ,\nu ,\mu )=\frac{1}{2}\lambda^{T}Q\lambda -\nu (\sum_{i=1}^{n}\lambda_{i}-1)-\sum_{i=1}^{n}\mu_{i}\lambda_{i},\\ Q=G^{T}G. \end{array}$$ (23)

where **1** is an all-ones vector, *v* is the Lagrange multiplier for the equality constraint, and $\mu_{i}$ is the Lagrange multiplier for the inequality constraint λ>=0. If $\lambda_{i}>0$, then $\mu_{i}=0$, indicating that the constraint is inactive; if $\mu_{i}>0$, then $\lambda_{i}=0$, meaning the variable is constrained to zero. This relationship is shown in Eq (24):

$$\mu_{i}\lambda_{i}=0,\forall i.$$ (24)

where *μ* is a penalty factor. When λ>0, it indicates that the task contributes and will not be subject to additional constraints;λ=0 means that the gradient of the task is sufficiently small at the current step and is temporarily excluded from optimization. Taking the partial derivative of Eq (24) with respect to *λ* and setting it to zero yields the Eq (25):

$$\nabla_{\lambda }ℒ=2Q\lambda -v-\mu =0.$$ (25)

Eliminating *v*, the equation simplifies to:

Qλ=v. (26)

Given that $1^{T}\lambda =1$, a linear system is constructed by combining Eq (26) and then *λ* solved. The final loss function is is given in Eq (27):

$$\lambda_{1}=\frac{\left(g_{\text{constitutive}}^{T}\;\cdot g_{\text{constructive}})-(g_{\text{class}}^{T}\;\cdot g_{\text{constitutive}}\right)}{\left(g_{\text{class}}^{T}\;\cdot g_{\text{class}})+(g_{\text{constitutive}}^{T}\;\cdot g_{\text{constitutive}})-2(g_{\text{class}}^{T}\;\cdot g_{\text{constitutive}}\right)},\;\lambda_{2}=\frac{\left(g_{\text{class}}^{T}\;\cdot g_{\text{class}})-(g_{\text{class}}^{T}\;\cdot g_{\text{constructive}}\right)}{\left(g_{\text{class}}^{T}\;\cdot g_{\text{class}})+(g_{\text{constructive}}^{T}\;\cdot g_{\text{constructive}})-2(g_{\text{class}}^{T}\;\cdot g_{\text{constructive}}\right)}.$$ (27)

## 4 Results

### 4.1 Dataset

To comprehensively validate the effectiveness of the proposed MBCS framework, this study adopts a hybrid evaluation strategy combining “public benchmarks + domain-specific datasets”. Three intent detection datasets with distinctive characteristics were selected (detailed information in Table 1):

- **SNIPS:** Proposed by Coucke et al. [31], the SNIPS dataset contains 16,000 English utterances across 7 vertical domains (e.g., weather queries, music control, calendar management). It is widely used in intent detection and slot filling research due to its broad domain coverage, fine-grained annotations, and low noise level.
- **ATIS:** Originating from the DARPA speech understanding project, the ATIS dataset is a standard benchmark for intent detection and slot filling in the air travel domain (e.g., flight inquiries, ticket booking). Its high domain specificity aligns well with the few-shot professional scenario requirements of this study.
- **Inspection Records Dataset:** Inspection Records Dataset: Constructed by the research team through manual annotation of Chinese inspection records in the import-export cargo inspection domain, this dataset supports tax risk identification in China’s customs clearance context. It covers four high-risk intent types (e.g., under-declaration, false origin reporting, abnormal commodity classification, and key customs monitoring targets). The dataset exhibits strong domain specialization and high annotation complexity due to its professional nature.

**Table 1:** Data collections.

Dataset	Train Set	Validation Set	Test Set	Intent Categories	Domain	Class Balance*
SNIPS	12084	1000	700	7	7	Balanced
ATIS	4477	500	886	22	1	Imbalanced
Inspection Records	300	100	300	4	1	Balanced

### 4.2 Experimental environment and parameter settings

The experiments were conducted on a system running Ubuntu 20.04.5 LTS and CUDA 11.2. The hardware configuration includes an Intel(R) Xeon(R) Gold 5218R CPU with a base frequency of 2.10 GHz, 80 cores, and 128 GB of RAM, along with an NVIDIA GeForce RTX A6000 GPU equipped with 48 GB of VRAM. The implementation was developed under the PyTorch (2.1.2 + cu118) framework.

The experimental parameters are shown in Table 2. The maximum sequence length (Max Length) is set to 128 for the two public datasets and to 512 for the private dataset. The “Epoch” field indicates the number of training iterations for the 5-shot, 10-shot, and full-training settings, respectively.

**Table 2:** Experimental parameters.

Hyperparameters	Values	Hyperparameters	Values
Hidden Size	768	Learning Rate	6e-5
Max Length	64(512)	Dropout	0.1
Epoch	70(30)(4)	Optimizer	Adam
Batch Size	16	Activation Function	Relu

Common metrics for evaluating the effectiveness of classification models include accuracy and F1-score. Considering that the number of samples per class in the test set is not fully balanced, the study uses the weighted F1-score to assess model performance. The formulas are shown in Eqs (28) and (29).

$$Accuracy=\frac{\overset{C}{\underset{i=1}{\sum }}TP_{i}}{N}$$ (28)

$$Weighted-F1=2\sum_{i=1}^{C}\frac{N_{i}}{N}\cdot \frac{Precision_{i}\times Recall_{i}}{Precision_{i}+Recall_{i}}$$ (29)

where $Precision_{i}=\frac{TP_{i}}{TP_{i}+FP_{i}}$,$Recall_{i}=\frac{TP_{i}}{TP_{i}+FN_{i}}$ ,*C* denotes the total number of classes, *TP*_*i*_ represents the number of true positives for the i-th class, *FP*_*i*_ refers to the number of false positives for the i-th class, and *FN*_*i*_ indicates the number of false negatives for the i-th class.

### 4.3 Comparative experiment

**Fig 2:**
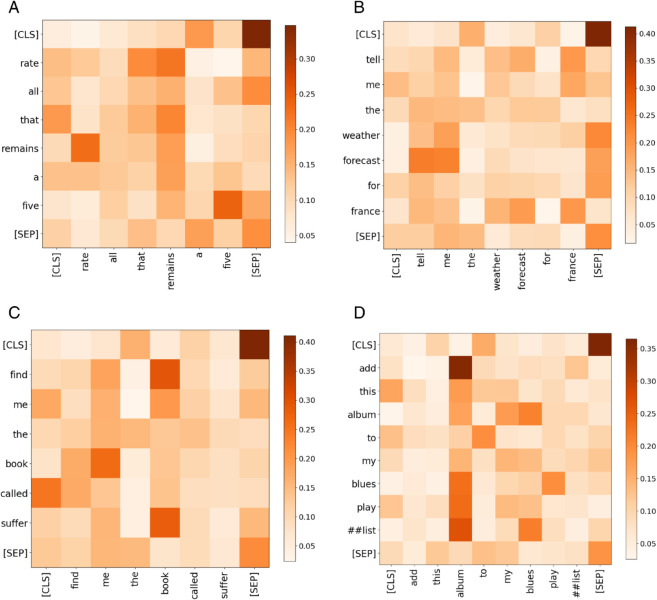
Heatmaps of self-attention matrix weights for different heads.

The study visualized the self-attention matrices of the same attention head across four sample sequences, as shown in Fig 2.

**Fig 3:**
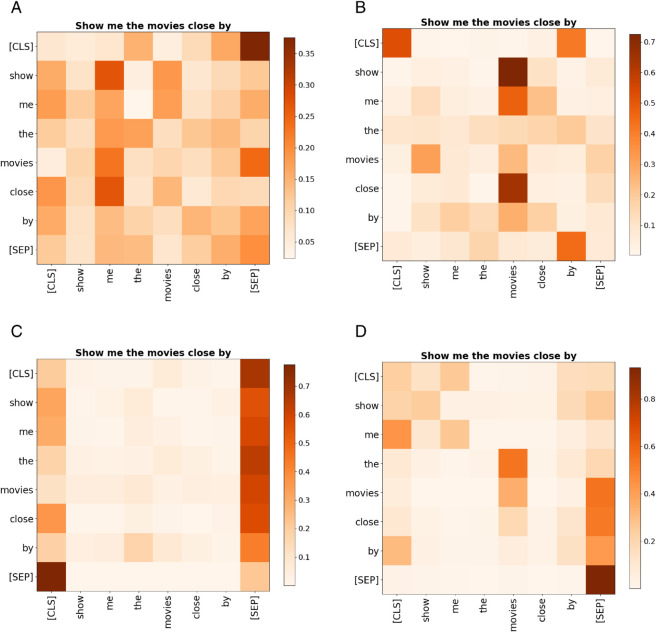
Heatmaps of self-attention matrix weights for different heads.

Fig 3 illustrates the attention weight scores computed by different heads. Taking the BERTbase model as an example, it consists of 12 encoder layers, each containing 12 attention heads. Each head corresponds to a self-attention matrix and independently computes attention scores, which reflect the correlations between different positions in the input sequence. Different heads focus on distinct aspects: some pay more attention to syntactic structures, while others emphasize semantic relationships.

Table 3 presents partial results of the synonym replacement strategy, using the SNIPS dataset as an example.

**Table 3:** Synonym replacement examples (SNIPS, Top-1 attention word).

Source Sentence	Target Sentence
Add this (album) to my blues playlist	Add this (record album) to my blues playlist
Show me the (movies) close by	Show me the (film) close by
tell me the (weather) forcast for france	tell me the (weather condition) for france
(Book) spot for three at Maid-Rite Sandwich	(Reserve) spot for three at Maid-Rite
Shop in Antigua and Barbuda	Sandwich Shop in Antigua and Barbuda

**Fig 4:**
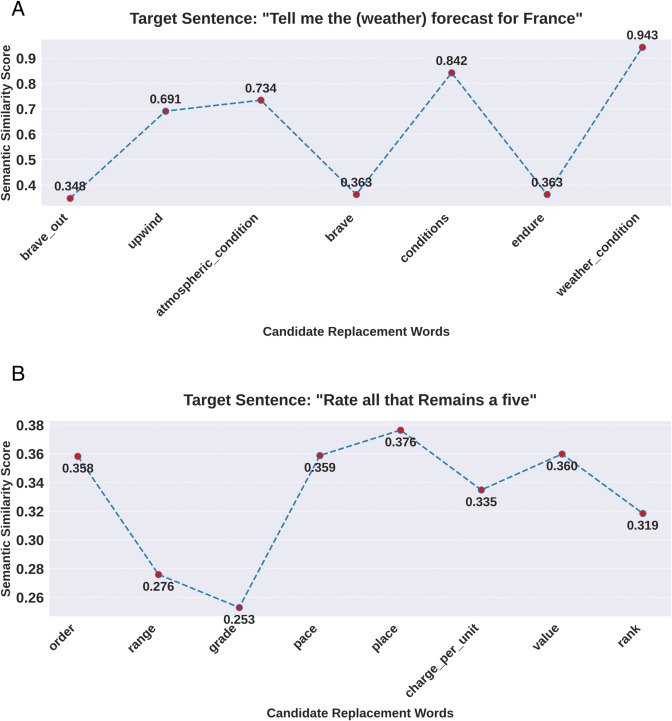
Semantic similarity comparison of candidate words.

Fig 4 visually compares the semantic similarity between partial synonyms and candidate words (including all parts of speech and contextual information).

**Fig 5:**
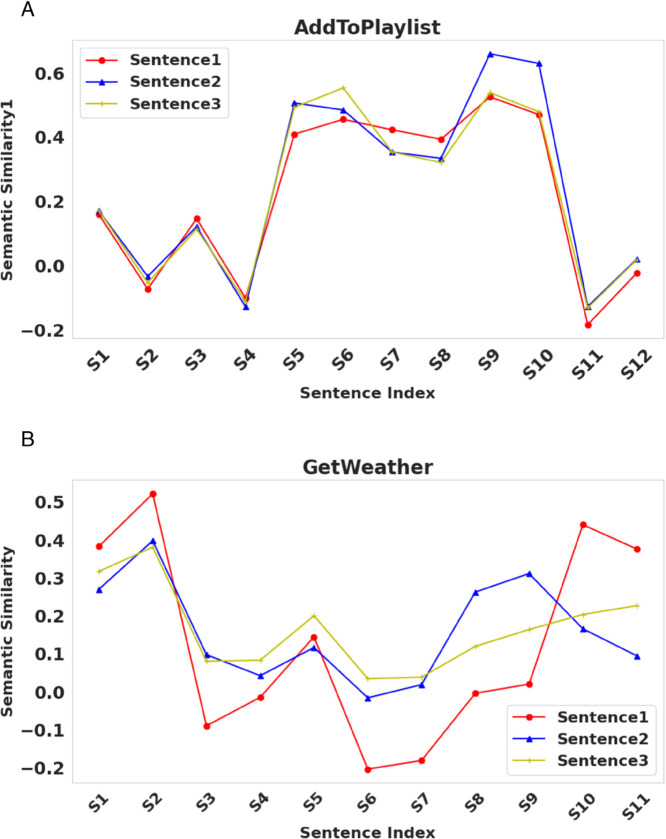
Semantic similarity comparison of candidate words.

The study conducted a comparative analysis of the semantic similarity between three sentences of the same category and sentences from other distinct categories, with the visual results presented in Fig 5. The figure clearly shows that the semantic similarity among sentences of the same category is significantly higher than that between different categories, intuitively reflecting the semantic consistency within categories and the distinctions between them.

To evaluate the performance of MBCS, the study conducted comparative experiments with baseline model selection. The results of MBCS were compared against those of the baseline model and several state-of-the-art models in few-shot intent detection. The findings demonstrate the superior performance of the MBCS model, with all experimental results reported on a percentage scale (Table 4).

**Table 4:** Comparative experiments with baseline models.

Method	BERT	FastText	T5
**SNIPS**	**98.43**	98.12	98.32
**ATIS**	96.11	95.83	**96.15**
**Inspection Records**	**90.52**	89.79	90.49
**Average**	**95.02**	94.58	94.99

The study evaluated the accuracy of three classic text classification models—FastText [32], T5 [33], and BERT—across three datasets. FastText is an efficient tool for text classification and word vector generation, proposed by the Facebook AI Research team in 2016. It combines a bag-of-words model with a shallow neural network, designed to handle large-scale text data efficiently while maintaining high classification accuracy. T5 (Text-to-Text Transfer Transformer), introduced by Google in 2019, is a pre-trained language model based on the Transformer architecture. It adopts the classic encoder-decoder structure and embodies the core idea of reformulating all natural language processing tasks into a unified “text-to-text” format, where both input and output are text sequences. This unified framework enables T5 to handle diverse NLP tasks—such as text classification, machine translation, question answering, and text summarization—in a generalizable manner (Table 5).

**Table 5:** Comparative experiments.

Acc.												
Method	SNIPS			ATIS			Inspection Records		
	5	10	full	5	10	full	5	10	30
BERT_base	81.43	87.89	98.43	81.09	85.71	96.11	70.17	80.97	90.52
InductNet	82.77	86.92	-	82.84	86.21	-	72.71	75.45	-
MBCS(ours)	**84.62**	**91.32**	**98.86**	**84.53**	**90.33**	**96.86**	**74.36**	**85.88**	**96.20**
**Weighted-F1**									
**Method**	**SNIPS**			**ATIS**			**Inspection Records**		
	5	10	full	5	10	full	5	10	30
BERT_base	78.21	85.34	96.87	77.85	83.26	94.45	66.52	79.31	88.27
InductNet	79.63	83.45	-	80.82	83.72	-	68.94	70.73	-
MBCS(ours)	**81.27**	**88.64**	**96.98**	**82.06**	**88.02**	**94.65**	**71.78**	**84.64**	**93.41**

The study compared the proposed approach with baseline and state-of-the-art few-shot text classification models across three datasets. InductNet [34] innovatively employs a dynamic routing algorithm within a meta-learning framework to learn generalized class-level representations from limited samples, significantly improving text classification performance in few-shot scenarios. Specifically, InductNet consists of three modules: an encoder module, an induction module, and a relation module. The encoder module uses a bidirectional LSTM combined with a self-attention mechanism to encode input texts into fixed-size vector representations. The induction module, the core component of the model, utilizes a dynamic routing algorithm to map sample vectors from the support set to class vectors. By iteratively adjusting the connection strength between samples and classes, it automatically encapsulates class-level semantic information while reducing noise caused by sample diversity. The relation module employs a neural tensor layer to compare the query vector with class vectors and outputs a relation score indicating the degree of match between the query text and the class. Experimental results demonstrate that this model outperforms existing advanced methods on multiple text classification datasets.

In 5-shot settings, MBCS achieved accuracy improvements of approximately 3.19%,3.44% and 4.19% across the three datasets, with F1-score gains of 3.06%, 4.21% and 5.26%, respectively. In 10-shot settings, accuracy increased by 3.43%, 4.62%, and 4.91% with F1-score gains of 3.30%, 4.76%, and 5.33%, respectively. Compared to InductNet, the accuracy of the proposed method was boosted by up to 1.85% in 5-shot and 10.43% in 10-shot scenarios.

### 4.4 Ablation study

The study systematically conducted ablation experiments on the aforementioned three datasets to evaluate the individual contributions of the data augmentation strategy (DA), the multi-task learning framework (Multi-task). This comparative setup was designed to isolate and assess the independent impact of data augmentation techniques on model performance. The experimental design followed a rigorous ablation approach, focusing specifically on evaluating the effects of data augmentation technology and the representation learning auxiliary task on model performance (Table 6).

**Table 6:** Ablation study (5-shot).

SNIPS.				
DA	Multi-task	Model	Acc.	F1-score
x	x	BERT_base_	81.43	78.21
✓	x	MBCS	83.28	80.87
x	✓	MBCS	82.54	80.20
✓	✓	MBCS	**84.62**	**81.27**
**ATIS.**				
**DA**	**Multi-task**	**Model**	**Acc.**	**F1-score**
x	x	BERT_base_	81.09	77.85
✓	x	BERT_base_	83.18	80.33
x	✓	MBCS	81.72	78.49
✓	✓	MBCS	**84.53**	**82.06**
**Inspection Records.**				
**DA**	**Multi-task**	**Model**	**Acc.**	**F1-score**
x	x	BERT_base_	70.17	66.52
✓	x	BERT_base_	73.56	70.02
x	✓	MBCS	72.04	68.89
✓	✓	MBCS	**74.36**	**71.78**

The experimental results indicate that the performance of the synonym replacement strategy is significantly correlated with the quality of the training set. To ensure the reliability and stability of the experimental outcomes, the study randomly sampled multiple distinct subsets from the original training set and conducted multiple independent experiments on each subset. By performing statistical analysis on the results, excluding outliers, and averaging the outcomes, the study minimized the impact of training set sampling bias, thereby enhancing the representativeness and persuasiveness of the findings.

The results demonstrate that:

(1) This study systematically evaluated the proposed method on three datasets: SNIPS, ATIS, and Inspections Records, by testing the effect of replacing 1 to 5 synonyms per sentence. Experimental results show that the performance of the synonym replacement strategy is closely related to the original sequence length: longer sequences generally require the replacement of more synonyms to achieve optimal results. It is worth noting that, unlike English texts, Chinese sequences possess a natural advantage under this strategy—synonym replacement rarely leads to grammatical distortion or semantic ambiguity. This characteristic contributes to a more pronounced performance improvement of the strategy on the Chinese Inspections Records dataset.

The data augmentation strategy based on synonym replacement yielded an accuracy improvement of at least 1.85% and up to 3.39% in the 5-shot scenario. This suggests that the method performs better on out-of-domain data than on in-domain data. By introducing synonym variations, the study effectively expanded the lexical diversity of training samples and enhanced the model’s generalization capability for linguistic expressions.

(2) The incorporation of the contrastive learning auxiliary task led to more substantial performance gains, improving accuracy by up to 1.87% and at least 1.11% in the 5-shot setting. This indicates that cross-task knowledge transfer through a shared representation space can effectively alleviate the data sparsity issue in few-shot scenarios. The study argues that the data augmentation strategy primarily enhances the model’s ability to recognize synonymous expressions, while the representation learning auxiliary task significantly improves its discriminative capacity for semantics across different classes.

## 5 Conclusions

To address the challenges of few-shot learning and domain adaptation in intent detection from manual inspection records within China’s customs import-export risk identification scenario, this study proposes MBCS, a few-shot intent detection model based on multi-task learning and semantics-constrained enhancement. The model first constructs a feature enhancement mechanism through a semantic similarity contrastive learning task, leveraging an adaptive loss function to dynamically balance the weights of the primary and auxiliary tasks, thereby effectively capturing domain-specific terminology and composite numeric encoding features. Next, it designs a semantics-constrained synonym replacement strategy that dynamically filters domain-adapted synonym sets to increase data diversity while preserving textual semantic integrity and business logic consistency. Finally, the enhanced features are fed into a classifier for intent detection.

Experiments on a dataset of import cargo inspection records demonstrate that, under 5-shot conditions, the proposed model improves accuracy and F1-score by 4.19%, and 5.26%, respectively, over baseline models. These results demonstrate that the model not only performs effectively in intent recognition for manual inspection records in China’s import-export supervision domain but also generalizes well to other vertical domains facing similar few-shot learning challenges.

Future work will focus on developing a quantifiable multi-dimensional semantic preservation evaluation framework and exploring attention distillation-based lightweight techniques to further enhance model efficiency and cross-scenario transfer capability.

## References

[1] Tesfagergish SG, Damasevicius R, Kapociute-Dzikiene J. Enhancing intent detection through ChatGPT-driven data augmentation. In: Doctoral Symposium on Computational Intelligence. 2023. p. 309–19.

[2] Huang S, Qin L, Wang B, Tu G, Xu R. SDIF-DA: a shallow-to-deep interaction framework with data augmentation for multi-modal intent detection. In: ICASSP 2024 - 2024 IEEE International Conference on Acoustics, Speech and Signal Processing (ICASSP). 2024. p. 10206–10. 10.1109/icassp48485.2024.10446922

[3] Xue J, Yin C, Li C, Bai J, Chen H, Rong W. Prompt based CVAE data augmentation for few-shot intention detection. In: International Conference on Knowledge Science, Engineering and Management. 2024. p. 312–23.

[4] Guo Z, Niu K, Chen X, Liu Q, Li X. Few-shot intent detection by data augmentation and class knowledge transfer. In: 2024 6th International Conference on Natural Language Processing (ICNLP). 2024. p. 458–62. 10.1109/icnlp60986.2024.10692688

[5] Dadas S, Protasiewicz J, Pedrycz W. A deep learning model with data enrichment for intent detection and slot filling. In: 2019 IEEE International Conference on Systems, Man and Cybernetics (SMC). 2019. p. 3012–8. 10.1109/smc.2019.8914542

[6] Li Y, Zhang X. Step-wise staged data augmentation for few-shot intent recognition. Computer Systems & Applications. 2023;32(1):406–12. doi: 10.15888/j.cnki.csa.008891

[7] Xu W, Zhou P, You C, Zou Y. Semantic transportation prototypical network for few-shot intent detection. In: Interspeech. 2021. p. 251–5.

[8] Snell J, Swersky K, Zemel RS. Prototypical networks for few-shot learning. In: Advances in Neural Information Processing Systems. 2017. p. 4077–87.

[9] Yang S, Du Y, Huang J, Li X, Du S, Liu J, et al. Few-shot intent detection with mutual information and contrastive learning. Applied Soft Computing. 2024;167:112338. doi: 10.1016/j.asoc.2024.112338

[10] Casanueva I, Temčinas T, Gerz D, Henderson M, Vulić I. Efficient intent detection with dual sentence encoders. In: Proceedings of the 2nd Workshop on Natural Language Processing for Conversational AI. 2020. 10.18653/v1/2020.nlp4convai-1.5

[11] Maqbool MH, Khan FA, Siddique AB, Foroosh H. Robust zero-shot intent detection via contrastive transfer learning. In: 2023 IEEE 17th International Conference on Semantic Computing (ICSC). 2023. p. 49–56. 10.1109/icsc56153.2023.00014

[12] Xin Y, Tinghuai M, Kexing P, Li J, Yongyi J. A meta-adapter integration learning method for few-shot scenarios across multiple domains. Journal of Computer Engineering & Applications. 2025;61(5). doi: j.issn.1002-8331.2309-0461

[13] Wang N, Cui S, Zhao E, Jin B. A classification method for online consultation on civil disputes based on deep transfer learning with small datasets. Engineering Applications of Artificial Intelligence. 2025;157:111360. doi: 10.1016/j.engappai.2025.111360

[14] Bhathiya HS, Thayasivam U. Meta learning for few-shot joint intent detection and slot-filling. In: Proceedings of the 2020 5th International Conference on Machine Learning Technologies. 2020. p. 86–92. 10.1145/3409073.3409090

[15] Bertinetto L, Henriques JF, Torr PHS, Vedaldi A. Meta-learning with differentiable closed-form solvers. In: International Conference on Learning Representations; 2019.

[16] Han J, Ding B, Li C. Meta-learning adversarial domain adaptation network for few-shot text classification. In: Findings of the Association for Computational Linguistics: ACL-IJCNLP 2021 . 2021. p. 1664–73.

[17] Bao Y, Wu M, Chang S, Barzilay R. Few-shot text classification with distributional signatures. In: International Conference on Learning Representations; 2020.

[18] Wang X, Du Y, Chen D, Li X, Chen X, Lee Y, et al. Constructing better prototype generators with 3D CNNs for few-shot text classification. Expert Systems with Applications. 2023;225:120124. doi: 10.1016/j.eswa.2023.120124

[19] Yang S, Du Y, Zheng X, Li X, Chen X, Li Y, et al. Few-shot intent detection with self-supervised pretraining and prototype-aware attention. Pattern Recognition. 2024;155:110641. doi: 10.1016/j.patcog.2024.110641

[20] Schick T, Schütze H. Exploiting cloze-questions for few-shot text classification and natural language inference. In: Proceedings of the 16th Conference of the European Chapter of the Association for Computational Linguistics: Main Volume. 2021. 10.18653/v1/2021.eacl-main.20

[21] Gao T, Fisch A, Chen D. Making pre-trained language models better few-shot learners. In: Proceedings of the 59th Annual Meeting of the Association for Computational Linguistics and the 11th International Joint Conference on Natural Language Processing (Volume 1: Long Papers). 2021. p. 3816–30. 10.18653/v1/2021.acl-long.295

[22] Zhang H, Zhang X, Huang H, Yu L. Prompt-based meta-learning for few-shot text classification. In: Proceedings of the 2022 Conference on Empirical Methods in Natural Language Processing. 2022. 10.18653/v1/2022.emnlp-main.87

[23] Zeng HLL, Lv SHZ. Prompt learning-driven news public opinion risk identification method. Journal of Computer Engineering & Applications. 2024;60(1). doi: 10.3778/j.issn.1002-8331.2208-0295

[24] Feng Z, Peng L, Dang K, Zhou M, Kuang P, Wu M, et al. TIPS: two-level prompt selection for more stability-plasticity balance in continual learning. Pattern Recognition. 2026;171:112276. doi: 10.1016/j.patcog.2025.112276

[25] Zhang L, Zhang L. An ensemble deep active learning method for intent classification. In: Proceedings of the 2019 3rd International Conference on Computer Science and Artificial Intelligence. 2019. p. 107–11. 10.1145/3374587.3374611

[26] Farfan-Escobedo JD, Lopes K, Dos Reis JC. Active learning approach for intent classification in Portuguese language conversations. In: 2021 IEEE 15th International Conference on Semantic Computing (ICSC). 2021. p. 227–32. 10.1109/icsc50631.2021.00048

[27] Xiang H, Shi J, Zhang T, Zhao X, Liu Y, Ma Y. PromptAL: sample-aware dynamic soft prompts for few-shot active learning. Knowledge-Based Systems. 2025;329:114354. doi: 10.1016/j.knosys.2025.114354

[28] Shim J, Kang S. Why does this query need to be labeled?: Enhancing active learning through explanation-based interventions in query selection. Expert Systems with Applications. 2025;290:128443. doi: 10.1016/j.eswa.2025.128443

[29] Devlin J, Chang MW, Lee K, Toutanova K. Bert: pre-training of deep bidirectional transformers for language understanding. In: Proceedings of the 2019 Conference of the North American Chapter of the Association for Computational Linguistics: Human Language Technologies. 2019. p. 4171–86.

[30] Khosla P, Teterwak P, Wang C, Sarna A, Tian Y, Isola P. Supervised contrastive learning. Advances in Neural Information Processing Systems. 2020;33:18661–73. doi: 10.48550/arXiv.2004.11362

[31] Coucke A, Saade A, Ball A, Bluche T, Caulier A, Leroy D, et al. Snips voice platform: an embedded spoken language understanding system for private-by-design voice interfaces. arXiv preprint 2018. https://arxiv.org/abs/1805.10190

[32] Joulin A, Grave E, Bojanowski P, Mikolov T. Bag of tricks for efficient text classification. In: Proceedings of the 15th Conference of the European Chapter of the Association for Computational Linguistics: Volume 2, Short Papers. 2017. p. 427–31.

[33] Raffel C, Shazeer N, Roberts A, Lee K, Narang S, Matena M, et al. Exploring the limits of transfer learning with a unified text-to-text transformer. Journal of machine learning research. 2020;21(140):1–67.34305477

[34] Geng R, Li B, Li Y, Zhu X, Jian P, Sun J. Induction networks for few-shot text classification. In: Proceedings of the 2019 Conference on Empirical Methods in Natural Language Processing and the 9th International Joint Conference on Natural Language Processing (EMNLP-IJCNLP). 2019. 10.18653/v1/d19-1403

